# Supplementary material for: Smooth Interpolating Curves with Local Control and Monotone Alternating Curvature
Source: Comput Graph Forum. 2022 Oct 6;41(5):25–38. doi: 10.1111/cgf.14600 (PMC9827861; doi:10.1111/cgf.14600)
Supplement: Supplementary file 1 — Supplement Material [file CGF-41-25-s001.zip › Local-Smooth-Interpolating-MonoCurvature/extern/clothoids/submodules/Utils/docs/api-cpp/file_format.cc.html]

File format.cc — Utils v1.2

### Navigation

- index
- toc
- Utils »
- File format.cc

# File format.cc¶

Contents

- Definition (`format.cc`)
- Includes
- Namespaces
- Classes
- Functions
- Variables

## Definition (`format.cc`)¶

- Program Listing for File format.cc

## Includes¶

- `fmt/format-inl.h`

## Namespaces¶

- Namespace detail

## Classes¶

- Template Struct basic\_data

## Functions¶

- Template Function detail::format\_float
- Function detail::locale\_ref::get< std::locale >

## Variables¶

- Variable instantiate\_format\_float

### Quick search

### Table of Contents

- rang - Colors for your Terminal
- Zstr
- C++ API

«
hide menu

menu
sidebar
»

### Navigation

- index
- toc
- Utils »
- File format.cc

© Copyright 2021, Enrico Bertolazzi.
Created using Sphinx 4.2.0.
